# Egg intake and cognitive function in healthy adults: A systematic review of the literature

**DOI:** 10.1016/j.jnha.2025.100696

**Published:** 2025-10-07

**Authors:** Nessmah Sultan, Nicole J. Kellow, Caroline J. Tuck, Edellyne Cheng, Clare MacMahon, Jessica R. Biesiekierski

**Affiliations:** a Department of Nutrition, Dietetics and Food, Monash University, 264 Ferntree Gully Rd., Notting Hill VIC 3168, Australia; b Department of Allied Health, Swinburne University of Technology, John St., Hawthorn VIC 3122, Australia; c School of Allied Health, Human Services, and Sport, La Trobe University, Plenty Rd., Bundoora VIC 3086, Australia; d Human Nutrition Group, School of Agriculture, Food and Ecosystem Science, The University of Melbourne, Grattan St., Parkville VIC 3010, Australia

**Keywords:** Eggs, Cognition, Dementia, Nutrition, Aging, Systematic review

## Abstract

**Background:**

Cognitive decline is a growing public health concern, particularly in aging populations. Eggs are a widely consumed, nutrient-dense food containing choline, phospholipids, tryptophan, and omega-3 fatty acids, which individually support cognitive processes such as memory, attention, and neurogenesis. While these individual nutrients have demonstrated benefits in supplementation studies, the cognitive effects of whole egg consumption are not well established. This systematic review aimed to evaluate the association between whole egg consumption and cognitive function in healthy adults.

**Methods:**

A systematic search of five electronic databases (Medline, Embase, CINAHL Plus, SCOPUS, and PsychInfo) was conducted from database inception through February 2025. Studies were included if they investigated whole egg intake in relation to cognitive outcomes in healthy adults. Risk of bias was assessed using tools appropriate to study design. Due to heterogeneity in study methods, outcomes were synthesised narratively. Cognitive outcomes were categorised into domains including global cognitive function, memory, executive function, language, processing speed, and dementia risk.

**Results:**

Eleven studies met the inclusion criteria: one pre-post intervention study, six prospective cohort studies, three cross-sectional studies, and one case-control study. Study populations were predominantly older adults and included >38,000 participants. Two studies reported a reduced risk of dementia or cognitive impairment associated with moderate egg consumption (approximately 0.5–1 egg per day), while one study found increased risk at high intake levels (Over 1 egg per day). Several studies showed improvements in memory, verbal fluency, or processing speed with moderate—but not high—egg intake. The pre-post study reported improved reaction time following eight weeks of daily egg consumption (2 eggs per day). Heterogeneity in exposure measurement and cognitive testing methods limited direct comparisons across studies.

**Discussion:**

Moderate whole egg consumption may be associated with improvements in cognitive outcomes in healthy adults, including reduced dementia risk and better memory performance. However, findings are inconsistent and limited by differences in study design, dietary assessment, and cognitive testing. Further well-controlled intervention studies are needed to determine optimal intake levels, explore mechanisms, and assess whether eggs can be integrated meaningfully into dietary strategies to support cognitive aging. (PROSPERO registration: 408532).

## 1 Introduction

Cognitive decline, defined as a measurable reduction in cognitive abilities such as memory, attention or executive function [1], is a growing global health concern. Estimates suggest that mild cognitive impairment affects 15.0–21.7% of adults aged 70–89 years globally [2]. Beyond its impact on individuals and families, cognitive decline contributes significantly to healthcare system burdens, with dementia-related expenditures alone accounting for approximately US$1.3 trillion worldwide in 2019 [3].

Risk factors for cognitive decline are multifactorial and include chronic disease and cardiovascular conditions, potentially due to the effects of long-term systemic inflammation, accumulation of abnormal proteins in neuronal tissue, impaired cerebral blood flow, and immune dysfunction [[4], [5], [6], [7]]. Diet is also a key modifiable factor influencing cognitive health. In particular, dietary patterns that emphasise plant-based foods and minimise intake of saturated fats and animal-derived foods, including the Mediterranean, DASH, and MIND diets, have consistently been associated with reduced risk of cognitive decline [[8], [9], [10]]. Building on this, recent research has turned to examining specific nutrient-dense foods that may offer neuroprotective benefits.

Eggs are a widely consumed, nutrient dense food that contain several compounds with potential neurocognitive benefits. Eggs contain protein (including the amino acid tryptophan), choline, and fat (including phospholipids, docosahexaenoic acid, linoleic acid), nutrients that influence neurotransmission, neurogenesis, and brain function. Protein (6.29 g per medium sized egg) has been linked to improved memory and reaction time in healthy young adults, and reduced risk of cognitive impairment in older cohorts [[11], [12], [13], [14]]. The essential amino acid tryptophan (77 mg per egg) crosses the blood brain barrier for conversion to the neurotransmitter serotonin, which is involved in decision-making and memory [15,16].

Moreover, choline (150 mg per egg) is a precursor to acetylcholine, a neurotransmitter involved in memory and learning [17,18]. Dietary choline intake between 187–399 mg per day has consistently been associated with improved cognitive performance across healthy younger and older adults [[19], [20], [21]]. Although these associations are observational and not always consistent across cognitive domains.

Eggs are also rich in fat (10.3 g per egg) mostly made up of phospholipids and unsaturated fatty acids [22]. Phospholipids (approximately 33% of egg lipids) modulate neurotransmitter receptors and have been linked to enhanced reaction time in clinical trials of healthy middle-aged men when given as a daily 13.5 g supplement for 3 weeks [23,24]. Additionally, docosahexaenoic acid (DHA, 0.5–1.0% of egg lipids) is an omega-3 fatty acid essential for neurodevelopment and neuroplasticity, and has been associated with a lower risk of dementia [[25], [26], [27], [28]]. Conversely, linoleic acid (LA, 11–16% of egg lipids) is an omega-6 fatty acid which may be disadvantageous for cognition and has been linked to increased risk of dementia in older adults and reduced mathematics test scores in school children [29,30]. LA can inhibit DHA activity in the brain, therefore the two fatty acids act in opposition to each other [30]. While these nutrients have demonstrated cognitive impacts when supplemented individually, less is known about the cognitive effects of whole egg consumption [30].

Despite their promising nutrient profile, few studies have directly examined the association between whole egg consumption - a widely accepted source of nutrients that can be easily incorporated into diverse dietary patterns - and cognitive function in healthy adults. Evidence to date has been limited and heterogeneous, with variability in dietary assessment methods, study designs, and cognitive testing tools. This systematic literature review (SLR) aimed to synthesise existing human studies investigating the relationship between habitual or supplemented whole egg intake and cognitive outcomes in adults, focusing on studies that either included only people without diagnosed metabolic or cardiovascular diseases (including type 2 diabetes, metabolic syndrome, cardiovascular disease, or chronic kidney disease), or statistically adjusted for chronic disease status in their analysis. It is a continuation of our previous systematic review, which evaluated egg consumption in relation to gastrointestinal outcomes [31], and examines whether neuroprotective benefits may be observed, thus informing the potential role of eggs in dietary strategies to mitigate age-related cognitive decline.

## 2 Methods

### 2.1 Eligibility criteria, databases, search strategy

This review was conducted according to the Preferred Reporting Items for Systematic Reviews (PRISMA) guidelines [32], and was registered with the Prospective Register of Systematic Reviews (PROSPERO) in March 2023 (408532).

Five electronic databases (Ovid Medline, Embase, CINAHL Plus, SCOPUS, and PsychInfo) were systematically searched from their inception until April 2023. An updated search was conducted in February 2025 to capture studies published between April 2023 and February 2025.

Eligible studies examined the association between whole chicken egg consumption (any quantity) and cognitive outcomes in adults without chronic disease (aged ≥18). Cognitive outcomes were defined as any measure of brain function, including global cognitive function, cognitive impairment, executive function, memory, decision-making, reaction-time, or neuronal activity. Studies were excluded if they: [1] involved animals or children [2], assessed non-whole egg components only (e.g. isolated egg whites or egg yolks), or [3] were review articles or case studies. Prospective cohort studies that included participants with chronic disease were included only if statistical models adjusted for disease status.

A research health sciences librarian was consulted to assist with development of the search strategy. The following key words and MeSH terms were used: (egg* adj3 (ingest* or intake or eat* or consum*) AND (exp Cognition/ or exp Learning/ or exp Thinking/ or exp Perception/ or exp Psychophysiology/ or exp Executive Function/ or inhibition or memory or interoception or decision-making or decision making or problem-solving or problem solving or reaction-time or reaction time or processing-speed or processing-speed or risk-taking or risk taking). Searches were restricted to human studies published in the English language. The full search strategy used is provided in Supplementary Table S1.

This systematic review was initially designed to examine the impact of whole egg consumption on both gastrointestinal and cognitive health outcomes. Due to the volume and diversity of findings, the review was divided into two manuscripts. The current manuscript focuses specifically on cognitive outcomes in healthy adults. The other manuscript focused on gastrointestinal outcomes and has been published [31].

### 2.2 Study selection and data extraction

Search results were imported into Endnote (version X9, Clarivate Analytics) for de-duplication. Remaining records were then uploaded to Covidence Systematic Review Software (Veritas Health Innovation, Melbourne, Australia) for screening. Title and abstract screening, followed by full-text review, were conducted independently by two reviewers (NS and JRB). Any disagreements were resolved through discussion or consultation with a third reviewer (CJT).

Data extraction was conducted following full-text screening. The following information was extracted from each study: author, year of publication, country of origin, study design, sample size, type of intervention or dietary exposure, cognitive outcomes examined, outcome measures used, key results, and funding source. One reviewer completed the initial data extraction (NS), which was independently verified by a second author (EC).

### 2.3 Data items

Extracted cognitive outcomes were categorised into the following domains based on how they were defined by the study authors: cognitive decline / global cognitive function, language functioning, verbal learning, memory, processing speed, decision-making, attention, executive function, risk-taking, reaction time, visuospatial ability, orientation, cognitive flexibility, and interoception. There was some overlap between categories, as studies often measured more than one cognitive outcome using one tool. For example, tools that measured ‘executive function’ tended to also measure ‘processing speed’. When this occurred, relevant data were extracted under each cognitive outcome separately. In this review, similarly-measured cognitive outcomes are reported together. The tools used to assess these outcomes were also documented. For cohort studies, information on covariates that were measured and controlled for in analyses, as well as statistical analysis methods were recorded.

### 2.4 Risk of bias assessment

Risk of bias assessment was assessed independently by two authors (NS and NJK), with disagreements resolved through discussion. One of four established tools was used, depending on study design. Pre-post intervention studies were assessed using the Cochrane Risk Of Bias In Non-Randomized Studies – of Interventions (ROBINS-I) [33], which evaluates seven domains: confounding factors, classification of intervention, participant selection, deviations from intended interventions, missing data, outcome measurement, and selection of reported results. Studies were rated as ‘no information’, ‘low risk’, ‘moderate risk’, ‘serious risk’ or ‘critical risk of bias’. Prospective cohort studies were assessed using the Cochrane Risk of Bias In Non-Randomized Studies-of Exposures (ROBINS-E) tool [34], which assesses seven domains: confounding factors, exposure measurement, participant selection, post-exposure interventions, missing data, outcome measurement, and selection of reported results. Ratings were classified as ‘no information’, ‘low risk’, ‘moderate risk’, ‘serious risk’ or ‘critical risk’. Cross-sectional studies were assessed using the Joanna Briggs Institute (JBI) Critical Appraisal Checklist for Cross-Sectional Studies [35], which evaluates eight domains including participant selection criteria, clear description of study subjects and setting, exposure measurement, objective measurement of the condition, identification and control of confounding factors, and statistical analyses. Case-control studies were evaluated using the JBI Critical Appraisal Checklist for Case Control Studies [35], which examines 10 domains including group comparability, exposure measurement, confounding control, and adequacy of the exposure period. For both JBI tools, risk of bias for each domain was classified as ‘yes’, ‘no’, ‘unclear’, or ‘not applicable’. A ‘yes’ rating indicates lower risk of bias.

### 2.5 Statistical analysis and narrative synthesis

Due to substantial heterogeneity in study design, dietary exposure measurement, cognitive outcome domains, and assessment tools, a meta-analysis was not conducted. Instead, a structured narrative synthesis was performed. Included studies were grouped by cognitive outcome domain and summarised in terms of study design, population characteristics, methods of assessing egg consumption and cognitive function, key findings, and risk of bias. Where possible, patterns of association between egg intake and cognitive outcomes were identified based on consistency of directionality and effect size across studies.

## 3 Results

### 3.1 Study selection

**Fig. 1:**
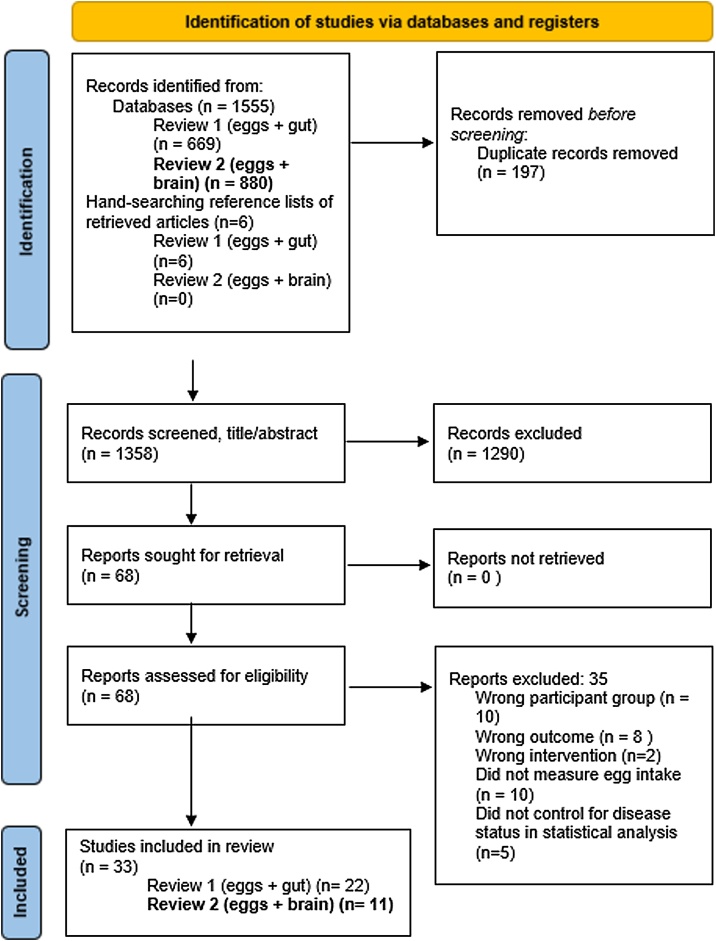
PRISMA flow diagram of study selection. Review 2 (eggs + brain) studies included in narrative synthesis (n = 11).

The primary database search conducted up to April 2023 identified 1,278 records, and an updated search in February 2025 identified a further 78 records. After removal of duplicates and initial screening, 68 articles were assessed for eligibility. Eleven studies met the inclusion criteria for the cognitive outcomes study and were included in this review. An additional five studies initially appeared to meet inclusion criteria, but were ultimately excluded from this review as they did not control for chronic disease status in their statistical analyses [2,[36], [37], [38], [39]]. The study selection process is summarised in the PRISMA flow diagram (Fig. 1).

### 3.2 Study characteristics

Study characteristics and key findings are reported in Table 1. The 11 included studies were published between 2016 and 2024 (data were collected between 1984–2020) and involved a total of 38,722 participants. Study designs comprised one pre-post intervention study [40], three cross-sectional studies [[41], [42], [43]], six prospective cohort studies [[44], [45], [46], [47], [48], [49]], and one case-control study [50]. The pre-post study was published as a randomised controlled trial which compared DHA-fortified to regular eggs, but in this review only within-group comparisons for the control group (regular eggs) were extracted. It was therefore considered a pre-post study [40]. Study populations spanned four countries: China (n = 6), USA (n = 3), Thailand (n = 1), and Finland (n = 1).

**Table 1:** Characteristics and key findings of included studies.

First author (year of publication)	Participants (country, no. of participants, age (years), BMI (kg/m^2^), sex)	Study Design			Covariates adjusted for	Cognitive assessment task	Cognitive sub-measures	Summary of findings
		Pre-post study						
		Design	Intervention	Duration				
Kaewsutas (2016)(40)	Thailand, n = 15 military males, 21.47 ± 0.19 y 22.95 ± 1.03 kg/m^2^, 0% females	Pre-post study. Results taken from a randomised controlled trial. Data extracted for control group pre-post analysis only	2 whole regular boiled eggs/d	8 weeks	N/A	a. visual computer-based “Go/NoGo” task performed at week 0, 4 and 8	a1. Reaction – time	a1. ↓ reaction time by 17%
							a2. False-alarm responses	a2. ∅
						b. Electroencephalograms (EEG) performed during Go/NoGo task	b. brain-wave frequencies categorised as Delta (<4 Hz), Theta (4−7 Hz), Alpha (8−13 Hz), Beta (13−30 Hz) and Gamma (30−42 Hz)	b. ↓ in Delta waves while completing NoGo task.

		Cross-sectional studies					
		Participant data sources/ recruitment	Measurement of egg intake	Covariates adjusted for	Cognitive assessment task	Cognitive sub-measures	Summary of findings
An (2022)(41)	USA, n = 2816 older adults, 68.98 (6.6) y, 53% females BMI reported in categories: <25 kg/m^2^: 26% 25 kg/m^2^ ≤30 kg/m^2^: 36% ≥30 kg/m^2^: 38%	Participants’ (aged ≥60) data obtained from the 2011−2012 and 2013−2014 National Health and Nutrition Examination Survey (NHANES)	Two 24 h recalls, first in-person (day 1), second by telephone (3−10 days later). Whole egg intake coded with the USDA Food and Nutrient Database for Dietary Studies (FNDDS). Whole egg consumers were defined as reporting egg consumption on both 24 h recall days (the average daily intake was 34.3 g of whole eggs)	Demographics: sex, age, BMI, race/ethnicity, education, marital status, poverty-income ratio, health insurance status, smoking status, alcohol use Medical history: chronic conditions Dietary intake: daily total energy intake	a. Consortium to Establish a Registry for Alzheimer’s Disease (CERAD)	a1. Verbal learning a2. Immediate memory a3. Delayed memory	a. ∅
					b. Animal Fluency	b.Verbal semantic fluency	b. ∅
					c. Digit Symbol Substitution Test	c1. Attention c2. Processing speed	c. ∅
Huang (2021)(42)	China, n = 4309, 68.4 y, BMI not reported, 55 females	Community-based Cohort Study on Nervous System Diseases (CCSNSD) 2018−2019	Validated 81-item semi-quantitative food frequency questionnaire (FFQ), measuring intake over previous 12 months. Median daily egg intake divided into quartiles, median (range): Q1: 6.3 (0.0−8.7) g Q2: 20.0 (15.4−21.7) g Q3: 42.9 (32.0−50.0) g Q4: 60.5 (60.0−81.8) g	Demographics: age, education, resident area, current employment, smoking, monthly household income, physical activity, sleep duration, alcohol intake Medical history: medical history, medication use Dietary intake: none	Montreal Cognitive Assessment (MoCA)	a. Cognitive impairment	a. Q4 egg consumers had a significantly ↑odds ratio for MCI compared to Q1 egg consumers (OR, 95% CI: 1.23, 1.03−1.48, p < 0.05)
						a1. Global cognitive function	a1. ∅
						a2. Memory	a2. ∅
						a3. Executive function	a3. ∅
						a4. Visuospatial	a4. Q2 and Q3 egg consumers had significantly ↑ scores compared to Q1, but ∅ when comparing Q4 to Q1
						a5. Language functioning	a5. Q2 egg consumers had significantly ↑ scores compared to Q1, but ∅ when comparing Q3 or Q4 to Q1
						a6. Attention	a6. ∅
						a7. Orientation	a7. ∅
Xu (2022)(43)	China, n = 7572, 83.2 ± 11.4 y, BMI not reported, 53% females Subgroup of participants with cognitive impairment: n = 900, 95 ± 8.5 y, 66% females	2018 Chinese Longitudinal Healthy Longevity Survey (CLHLS), a national prospective cohort study of Chinese adults aged 65 years and older.	Participants during interviews were asked to report their frequency of intake of specific food items. Egg intake categorised as: “daily or almost daily”, “not daily, but at least once per week”, “not weekly, but at least once per month”, “not monthly, but occasionally” “rarely or never”.	Demographics: sex, age, years of schooling, rurality, BMI, physical exercise, marital status, sleep duration, smoking status, living status, alcohol consumption Medical history: diabetes and hypertension Dietary intake: none	Mini mental state examination (MMSE)	Cognitive impairment. Cut-off scores: MMSE ≤ 17 for 0 years education; MMSE ≤ 20 for 1−6 years education; MMSE ≤ 24 for 7 or more years of education	∅

		Prospective cohort studies						
		Participant data sources/ recruitment	Measurement of egg intake	Duration (sample collection timepoints)				
An (2019)(44)	China, n = 2514 Age, BMI and sex divided based on dietary cholesterol intake quartiles: Q1 (<188 mg/day): 58 [56,62] years, 24.6 (22.9, 26.7) kg/m^2^, 56% females Q2 (188−283 mg/day): 59 [55,62] years, 24.6 (22.6, 26.6) kg/m^2^, 57% females Q3 (283−385 mg/day): 59 [55,62] years, 24.4 (22.6, 26.4) kg/m^2^, 57% females Q4 (>385 mg/day): 59 [55,62] years, 24.5 (22.6, 26.5) kg/m^2^, 46% females	Participants aged 50−70 from the Effects and Mechanism investigation of Cholesterol and Oxysterol on Alzheimer’s disease (EMCOA) study 2014	Unvalidated 33-item FFQ completed at baseline measuring habitual intake over previous year. Eggs measured as g/day. Egg intake quartiles: Q1: <21 g/day Q2: 21−45 g/day Q3: 45−60 g/day Q4: >60 g/day	Data collected at baseline and at 2.3 year follow up	Demographics: age, sex, years of education, BMI, smoking and drinking status Medical history: diabetes, hypertension and cardiovascular history, use of lipid-lowering medication Dietary intake: energy, protein, carbohydrates, fat, cholesterol, SFA, PUFA and MUFA.	a. MoCA	a. Cognitive impairment	a. ∅
						b. Auditory Verbal Learning Test Symbol	b. Memory: immediate recall; short recall; long recall	b. ∅
						c. Digit Modalities Test	c. Processing speed	c. ∅
						d. Wechsler Memory Scale – Revised		
						d1. Logical Memory Test	d1. Attention	d1. ∅
						d2. Digit span forwards	d2. Executive function	d2. ↓ executive function with increasing egg intake (β = −0.000022, p = 0.008)
Bishop (2019)(45)	USA, n = 3835, 74.32 ± 8.82 years, 56% females BMI reported as categorical: <18.5 kg/m^2^: 1.6% 18.5−25 kg/m^2^: 30.7% 25−30 kg/m^2^: 37.9% >30 kg/m^2^: 29.8%	Participants aged ≥65 years, from the 2012 and 2014 Health and Retirement Study (HRS) and the 2013 Health Care and Nutrition Study (HCNS)	Validated FFQ completed at 2013 follow up, measuring habitual intake over previous 12 months. Egg intake categorised as: no egg consumption, ≤1 serving/week, 2−6 servings/week, ≥7 servings/week Egg intake also categorised into quartiles: Q1: 0 eggs/d, (0 g/d) Q2: 0.09 eggs/d (3.87 g/d), Q3: 0.49 eggs/d (21.07), Q4: 1.36 eggs/d (58.48 g/d)	Cognition data collected at baseline (2012) and 2 year follow up (2014)	Demographics: age, sex, race/ethnicity, marital status, retirement status, years of education, longest occupational tenure, log-transformed household income and assets, BMI, physical activity, current smoking status, alcohol consumption Medical history: diagnosed chronic health conditions, disability, prior memory-related disease diagnosis Dietary intake: none	a. Telephone Interview of Cognitive Status (TICS)	a. Global cognitive function	a. ∅
						b. Immediate word recall	b & c. Memory	b. ∅
						c. Delayed word recall		c. ∅
						d. Serial 7 subtraction test	d & e. Executive function & Processing speed	d. ∅
						e. Backward counting task		e. ∅
Lee (2021)(46)	USA, n = 470, 63% females Median age and BMI divided according to egg intake: Low egg intake: 68.18 (11.14) y, 25.85 (4.75) kg/m^2^ High egg intake: 68.58 (11.26) y, 27.75 (6.01) kg/m^2^	Participants aged ≥50 years, from the 2002−2007 Adventist Health Study-2 (AHS-2) and the 2006−2007 AHS-2 Biopsychosocial Religion and Health Substudy (BRHS)	Validated 200-item quantitative FFQ, measuring habitual intake over previous 12 months. Egg intake categorised as: Low: <23 g/week Intermediate: 24−63 g/week High: >63 g/week	Data collected at baseline and 3.3 y follow up	Demographics: age, sex, race, education, BMI Medical history: cardiovascular disease risk score (hypertension, hyperlipidaemia, diabetes), depressive symptoms Dietary intake: total energy intake, dietary intake of fish, meat, dairy, fruit and vegetables	California Verbal Learning Test (CVLT)	Verbal learning & Memory	∅ in cross -sectional analysis of egg intake and CVLT performance ↓ rate of memory decline comparing intermediate egg intake vs low egg intake (β = 0.018, SE = 0.009, p = 0.043). But ∅ when comparing high egg intake to low egg intake (β = 0.011, SE = 0.009, p = 0.195)
Li (2022)(47)	China, n = 9028, 68.7 ± 7.0 y, 51% females BMI reported as categorical: <18.5 kg/m^2^: 5.2% 18.5−24 kg/m^2^: 54.7% ≥24 kg/m^2^: 40.1%	Participants aged ≥60 y old without cognitive impairment at baseline from the 2014−2020 Zhejian Ageing and Health Cohort Study.	Questionnaire at baseline interview asked about frequency and quantity of egg consumption in 2 questions: “how many days did you have eggs every week generally in the last months?” “how many eggs did you have in the days you consumed eggs?” Egg intake categorised as: none or not weekly 0.1−2.9 eggs/week 3.0–5.9 eggs/week ≥6.0 eggs/ week. Frequency of egg intake further categorised as: none or not weekly, 1−2 d/week ≥3 d/week. Quantity of egg intake was recoded as: 0.1−1.9 eggs/d, ≥2.0 eggs/d.	Data collected at baseline and 6 year follow up	Demographics: age, sex, race, education level, marital status, family income, BMI, smoking, alcohol, exercise, Patient Health Questionnaire-9 Scale (PHQ-9) Medical history: hypertension, diabetes, coronary heart disease Dietary intake: tea drinking, vegetable intake, fruit intake, red meat intake, fish intake, nuts intake	MMSE	Cognitive impairment. Cut off scores: MMSE of 17/18 for lower than primary education; MMSE 20/21 for primary education; MMSE 24/25 for higher than primary education	↓ risk of cognitive impairment in people who ate eggs 1−2 d/week compared to those who ate never or not weekly (none or not weekly vs ≥2 eggs/d 1−2days/week, RR (95% CI): 0.78 (0.67−0.91), p < 0.01). But ∅ when comparing highest egg intake to lowest (none or not weekly vs eating ≥2eggs/day ≥3 days/week, RR (95% CI): 0.94 (0.83–1.07), p = 0.38)
Sukik (2022)(48)	China, n = 4852, 53% females Age and BMI divided according to egg intake quartiles: Q1: 64.3 ± 7.9 y, 22.2 ± 3.5 kg/m^2^ Q4: 64.0 ± 7.6 y, 23.9 ± 3.4 kg/m^2^	Participants aged ≥55 years between 1991 and 2006 from the 1989−2015 China Health and Nutrition Survey (CHNS)	24 h dietary recall via interview over 3 consecutive days collected at baseline. Egg intake quartiles: Q1: none Q2: 1−20 g/d Q3: 21−50 g/d Q4: ≥51 g/d	Data collected at baseline (1991) and 15 y follow up (2006)	Demographics: age, sex, smoking, alcohol drinking, income, urbanisation, education, physical activity, BMI Medical history: hypertension Dietary intake: energy intake, intake of fat, total protein intake (without eggs).	a. TICS	a. Global cognitive function	a. ↑ cognitive function in Q4 egg consumers compared to Q1 (regression coefficient (95% CI): 0.96 (0.47, 1.45), p < 0.001)
						b. Self-reported memory rated on a scale	b. Memory	b. Ø
						c. Self-reported memory change rated on a scale	c. Memory decline	c. ↓ memory decline in Q4 egg consumers compared to Q1 (regression coefficient (95% CI): 0.81 (0.66, 0.99), p = 0.0015)
Ylilauri (2017)(49)	Finland, n = 2497, 0.0% females Age and BMI divided according to egg intake quartiles: Q1: 53.6 ± 5.2 y, 27.0 ± 3.7 kg/m^2^ Q4: 52.8 ± 5.0 y, 26.8 ± 3.5 kg/m^2^ Subgroup analysis (cognitive task completion at 4 y follow up): n = 480	Male participants aged 42−60 years in 1984−1989 from the 1984−1993 Kuopio Ischaemic Heart Disease Risk Factor Study (KIHD)	4-d guided food record, including 1 weekend using household measures, collected at baseline. Egg intake included eggs that were in mixed dishes and recipes as well as whole eggs. Egg intake quartiles: Q1: <14 g/day Q2: 14−25 g/d Q3: 26−43 g/d Q4: >44 g/d Subgroup analysis (cognitive task completion at 4 y follow up): Egg intake tertiles: T1: <16 g/d T2: 16−32 g/d T3: >32 g/d	Data collected at baseline (1984−1989) and 4 y later (1991−1993) Data relating to incident of dementia collected in 2014 (30 y follow up)	Demographics: age, examination year, education years, smoking, BMI, physical activity, alcohol intake Medical history: diabetes, coronary artery disease, lipid-lowering medication use, history of stroke, hypertension, blood glucose, serum long-chain n-3 PUFAs Dietary intake: energy intake, fruit, berry and vegetable intake, carbohydrate intake, fiber intake, cholesterol intake, coffee intake	a. Incidence of dementia	a. Incidence of dementia	a. ↓ risk of incident dementia in Q4 egg intake (HR (95% CI): 0.74 (0.53, 1.02), p = 0.04); but ∅ when cholesterol intake was adjusted for (0.78 (0.51, 1.19), p = 0.20)
						b. MMSE	b. Cognitive impairment	b. ∅
						c. Verbal Fluency Test	c. Language functioning	c. ↑ performance in T3 egg consumption (p = 0.02)
						d. Selective Reminding Test	d. Short and long-term memory	d. ∅
						e. Visual Reproduction Test	e. Visual memory	e. ∅
						f. Trail Making Test	f1. Processing speed f2. Attention f3. Executive function	f. ↑ performance in T3 egg consumption (p = 0.03)

		Case control studies						
		Participant groups	Measurement of egg intake	Duration				
Igbinigie (2024)(50)	China, n = 466, 73.6 ± 9.5 y, BMI not reported, 64% females	All participants: aged >50 y, attended community health service clinics between July-October 2020 Case: n = 233, people diagnosed with dementia by doctors at Huiai Hospital Control: n = 233, people who were not diagnosed with dementia	Face to face interviews with participants or their family members. Asked about frequency of egg consumption over the previous 2 years. Egg intake categorised as: Non-consuming or less than once a month, ≥Monthly and < Once a week (i.e., monthly), ≥Weekly and < Once a day (i.e., weekly), ≥Daily and < Twice a day (i.e., daily), ≥Twice a day.	N/A	Demographics: age, sex, education, family income, marital status, smoking, alcohol consumption Medical history: CVD score (hypertension, high blood cholesterol, diabetes, coronary heart disease and stroke), kidney disease, chronic bronchitis, head hurt, Parkinson’s disease, depression Dietary intake: red meat consumption (pork, beef and lamb), poultry, fish, vegetable and fruit consumption	Risk of dementia	Risk of dementia	↓ risk of dementia with higher frequency of egg intake. ‘daily’ intake had a lower OR compared to monthly intake (OR, 95% CI: 0.21, 0.08−0.53, p = 0.001) and weekly intake (0.48, 0.25−0.91, p = 0.024)

Continuous data presented as mean ± SD where parametric and median (IQR) where non-parametric. Only cognition-related outcomes reported across studies. BMI, body mass index; ∅, no change; ↑, increased; ↓, reduced; EEG, electroencephalograms; FFQ, food frequency questionnaire; CERAD, Consortium to Establish a Registry for Alzheimer’s Disease; MoCA, Montreal Cognitive Assessment; MMSE, Mini mental state examination; TICS, Telephone Interview of Cognitive Status; CVLT, California Verbal Learning Test.

Sample sizes ranged from 15 to 9028. With the exception of the pre-post study, which recruited male young adults, most studies focused on older adults. Participants in the observational studies had a mean age of 68 y (ranged between 50–80 y). Most studies included both male and female participants (average 50% female; n = 19, 046), however two studies enrolled only men [40,49].

There was a high level of heterogeneity across studies regarding measurement and categorisation of the exposure (egg consumption). The pre-post study provided 2 whole regular eggs per day for 8 weeks [40]. All observational studies (n = 10) collected data relating to habitual egg consumption. Some studies (n = 4) used food frequency questionnaires (FFQs), ranging from 33 to 200 food items, that measured food (including egg) consumption over the previous 12 months [42,[44], [45], [46]]. The 33-item FFQ used in one study was unvalidated [44]. Three studies reported using interviews to gather data on frequency of egg intake [43,47,50]. Two studies used 24 h recalls collected over 2–3 days [41,48], while one used 4-day guided food records which included one weekend day [49]. Across the studies, daily egg intake ranged from 0 to 82 g per day, or 0 to >6 eggs per week. Studies divided egg intake into tertiles/quartiles or categorised frequency of intake to compare outcomes.

Cognitive outcomes varied across studies; the most frequently used assessment tools included the mini-mental state examination (MMSE), used in 3 studies [43,47,49], Montreal Cognitive Assessment (MoCA), used in 2 studies [42,44], and Telephone Interview of Cognitive Status (TICS) used in 2 studies [45,48].

### 3.3 Risk of bias

**Fig. 2:**
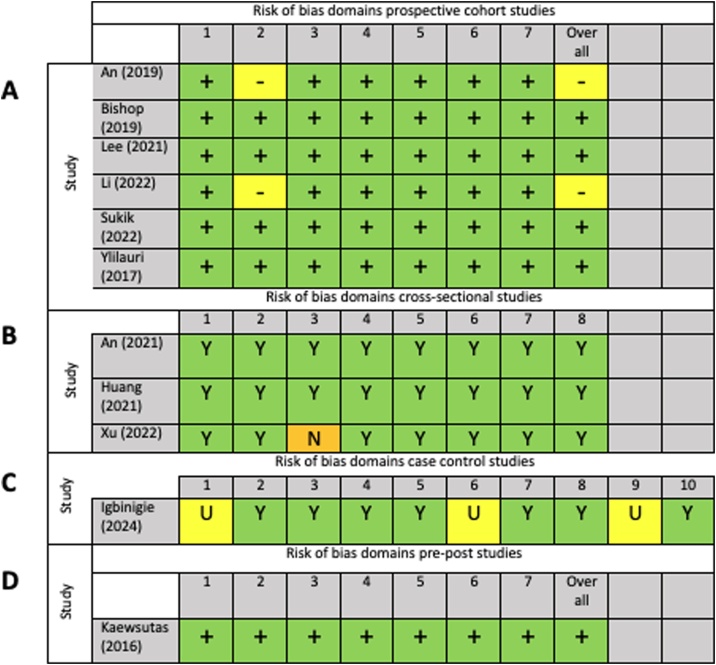
Risk of bias assessments (A) ROBINS-E [[44], [45], [46], [47], [48], [49]], (B) JBI for cross sectional studies [[41], [42], [43]], (C) JBI for case control studies [50], (D) ROBINS-I [40]. For ROBINS-E: D1, Bias due to confounding; D2, Bias arising from measurement of exposure; D3, Bias in selection of participants into the study (or into the analysis); D4, Bias due to post-exposure interventions; D5, Bias due to missing data; D6, Bias arising from measurement of the outcome; D7, Bias in selection of reported result. For JBI for cross-sectional studies:: 1,Were the criteria for inclusion in the sample clearly defined; 2, Were the study subjects and the setting described in detail; 3, Was the exposure measured in a valid and reliable way; 4, Were objective, standard criteria used for measurement of the condition; 5, Were confounding factors identified; 6, Were strategies to deal with confounding factors stated; 7. Were the outcomes measured in a valid and reliable way; 8, Was appropriate statistical analysis used. For JBI for case control studies: 1. Were the groups comparable other than the presence of disease in cases or the absence of disease in controls; 2. Were cases and controls matched appropriately; 3. Were the same criteria used for identification of cases and controls; 4. Was exposure measured in a standard, valid and reliable way; 5. Was exposure measured in the same way for cases and controls; 6. Were confounding factors identified; 7. Were strategies to deal with confounding factors stated; 8. Were outcomes assessed in a standard, valid and reliable way for cases and controls; 9. Was the exposure period of interest long enough to be meaningful; 10. Was appropriate statistical analysis used. ROBINS-I: D1, Bias due to confounding; D2: Bias due to classification of intervention; D3: Bias in selection of participants into the study (or into the analysis); D4: Bias due to missing data; D5: Bias due to deviations from intended intervention; D6: Bias due to outcome measurement; D7: Bias due to selection of reported results. Green “+/Y” = low risk of bias, yellow “−/N” = moderate risk of bias (For interpretation of the references to colour in this figure legend, the reader is referred to the web version of this article).

As presented in Fig. 2, most prospective cohort studies reported a ‘low’ risk of bias, according to the ROBINS-E criteria (n = 4), except for two [44,47], which were both classified as having ‘some concerns’ as participant egg intake was not estimated using a validated tool. Most cross-sectional studies were classified as ‘yes’ for all criteria (signifying a low risk of bias), but one study did not meet the requirements for criteria 3 (‘was the exposure measured in a valid and reliable way?’), as this study utilised an unvalidated method to measure habitual egg intake [43]. The sole case control study [50] was classified as ‘unclear’ under criteria 1 (‘were the groups comparable other than the presence of disease in cases or the absence of disease in controls?), as participant groups reported significant differences in several parameters, including age, education level, family income, marital status, and dietary intake, among others. However, this study controlled for these parameters in statistical analysis. This study was also marked as ‘unclear’ under criteria 6 (‘were confounding factors identified?’), as they did not control for total energy intake, and criteria 9 (‘was the exposure period of interest long enough to be meaningful?’), as the study compared incidence of dementia and only quantified habitual dietary intake over the previous 2 years. Lastly, the pre-post study [40] was found to have a ‘low’ risk of bias according to the ROBINS-I, as it was part of a randomised controlled trial. The funding sources of included studies are presented in Supplementary Table S2. Funding sources were free from bias as most studies were funded by scientific research institutions. Three studies were funded by the American Egg Board [41,45,46], which was not involved in the interpretation of findings. One study did not receive any funding [50].

### 3.4 Synthesis of results

#### 3.4.1 Cognitive impairment

Six studies examined associations between egg consumption and cognitive impairment, risk or incidence of dementia [[42], [43], [44]^47^,^49^,^50^].

One cross-sectional and one cohort study used the MMSE [43,47] to assess cognition. Xu et al. [14] reported no association between egg consumption and cognitive impairment when comparing regularity of egg intake (‘daily’ to ‘rarely or never’). Li et al. [51] found participants consuming 2 eggs/day for 1−2 days/week had a lower risk of impairment compared to rare consumers (never or not weekly) (RR (95% CI): 0.78 (0.67−0.91), p < 0.01). No significant association between egg consumption and risk of impaired cognitive function was observed in the highest egg intake group (≥2 eggs/day on ≥3 days/week; RR (95% CI): 0.94 (0.83–1.07), p = 0.38) [47].

Two cohort studies used the MoCA to assess cognition. Huang et al. [42] reported a higher risk of cognitive impairment in the highest quartile of egg consumption compared to the lowest (Q1 6.3 (0.0−8.7) g/day vs Q4 60.5 (60.0−81.8) g/day, OR (95% CI), 1.2 (1.03−1.48), p < 0.05). An et al. [44] reported no significant association with risk of cognitive impairment across quartiles of egg intake (Q1 < 21 g/day vs Q4 > 60 g/day HR (95% CI): 1.04 (0.81–1.33) p = 0.79).

One prospective cohort study examined incidence of dementia in men in Finland [49], and found a significantly lower risk of incident dementia in people in the highest quartile of egg intake compared to the lowest (Q4 > 44 g/day vs Q1 < 14 g/day, HR (95% CI), 0.74 (0.53, 1.02), p = 0.04). Notably, there was no association after cholesterol intake was adjusted for (HR (95% CI), 0.78 (0.51, 1.19), p = 0.20).

One case control study explored risk of dementia [50] and found that daily egg consumption was associated with lower odds of dementia compared to monthly consumption (‘daily’ vs ‘monthly’, OR (95% CI), 0.21 (0.08, 0.53), p = 0.001).

#### 3.4.2 Global cognitive function

Four studies measured the association between egg intake and global cognitive function in dissimilar cohorts and contexts. Huang et al. [42] (MoCA), Bishop and Zuniga [45] (TICS), and Ylilauri et al. [49] (MMSE) did not find any association between egg intake and global cognitive function. However, Sukik et al. [48] reported better cognitive function using the TICS in the highest egg consumers compared to the lowest (Q1 0 g/day vs Q4 ≥ 51 g/day, p < 0.001) in a cohort of Chinese adults aged >55 years.

#### 3.4.3 Verbal learning and memory

Seven studies explored the association between egg consumption and verbal learning or memory outcomes [41,42,[44], [45], [46]^48^,^49^]. Five studies reported no significant associations between egg intake and measures of learning or memory [41,42,44,45,49]. Two studies reported significant findings. Lee et al. [46], using the California Verbal Learning Test (CVLT), found a reduced rate of memory decline comparing intermediate egg intake to low intake (<23 g/week vs 24−63 g/week, β = 0.018, SE = 0.009, p = 0.043) in a 3.3 year follow up of people aged ≥50 years in the USA. No difference was observed between high and low intake groups (<23 g/week vs >63 g/week, β = 0.011, SE = 0.009, p = 0.195). Sukik et al. [48] reported a lower rate of self-reported memory decline in participants in the highest quartile of egg consumption compared to the lowest (Q1 0 g/day vs Q4 ≥ 51 g/day, p = 0.0015) in Chinese adults aged >55 years.

#### 3.4.4 Language functioning/Verbal fluency

Three studies explored the link between habitual egg consumption and language functioning or verbal fluency [41,42,49]. An et al. [41] did not find an association between egg intake and verbal semantic fluency in older adults in the USA. Ylilauri et al. [49] reported that Finnish men in the highest tertile for egg intake performed better on the Verbal Fluency Test compared to those in the lowest tertile (T1 < 16 g/day vs T3 > 32 g/day; median (IQR) scores: 31.0 (29.2, 32.8) vs 34.1 (32.3, 35.9) p = 0.02, adjusted for covariates). Huang et al. [42] found that participants in the second quartile of egg intake had significantly higher scores on the language subdomain of the MoCA compared to those in the lowest quartile (Q1 6.3 (0.0−8.7) g/day vs Q2 20.0 (15.4−21.7) g/day, p = 0.046). However, no significant differences were observed in the third (Q3 42.9 (32.0–50.0) g/day) or fourth quartiles (Q4 60.5 (60.0–81.8) g/day) compared to the first.

#### 3.4.5 Processing Speed/ executive Function/Attention

Although considered distinct cognitive domains, processing speed, executive function and attention were measured using overlapping tools across studies. Therefore, results for these domains are presented together from five studies assessing these domains [41,42,44,45,49]. Most found no associations with egg consumption. An et al. [44] reported a significant inverse linear relationship between egg intake and executive function as measured by the Digit Span Forwards test (p = 0.008). Ylilauri et al. [49], using the Trail Making Test to assess processing speed, attention, and executive function, reported better performance among participants in the highest tertile of egg intake compared to the lowest (T1 < 16 g/day vs T3 > 32 g/day, p = 0.03)

#### 3.4.6 Visuospatial ability and orientation

Huang et al. [42] assessed visuospatial ability and orientation using subdomains of the MoCA. Participants in the second and third quartiles of egg intake performed significantly better than those in the lowest quartile on the visuospatial domain (Q1 6.3 (0.0−8.7) g/day vs Q2 20.0 (15.4−21.7) g/day, p < 0.001; Q1 vs Q3 42.9 (32.0−50.0) g/day, p = 0.010). No difference was observed between the first and fourth quartiles (p = 0.877). There were no differences in orientation scores across quartiles of egg consumption.

#### 3.4.7 Reaction-time and brain wave activity

One study [40] assessed reaction time, false-alarm responses, and brain wave activity in a pre-post trial involving 15 healthy young adult males in the Thai military. Participants consumed two regular eggs per day for 8 weeks and exhibited a 17% improvement in reaction-time and reductions in Delta brain wave frequency, but no change in false-alarm responses.

## 4 Discussion

This systematic review synthesised evidence from 11 studies investigating the relationship between whole egg consumption and cognitive function in healthy adults. The findings were heterogeneous, with some studies reporting no association [41,[43], [44], [45]], while others indicated benefits for specific cognitive domains—particularly verbal memory, verbal fluency, reaction-time, and reduced risk of cognitive impairment or dementia [40,[46], [47], [48], [49], [50]]. Overall, the review suggests that moderate egg intake may be associated with cognitive benefits in some domains, although substantial heterogeneity in methods and outcome measures limits strong conclusions.

Cognitive impairment and dementia risk were the most consistently investigated outcomes. Two prospective cohort studies found that individuals with moderate egg consumption had a lower risk of cognitive impairment or dementia compared to those with low intake [47,49]. In both cases, the protective effect was observed in moderate but not high intake categories, suggesting a possible threshold beyond which benefits may either be not observed, or may even be detrimental. These findings are consistent with previous observational studies indicating that dietary choline intake between 187–399 mg per day (equivalent to approximately two eggs) is associated with better cognitive function, with a non-significant effect seen in excess of 400 mg choline per day [20].

In contrast, a cross-sectional study by Huang et al. [42] reported an increased risk of cognitive impairment in the highest egg intake group. This discrepancy may reflect methodological differences. Huang et al. [42] relied on MoCA subdomains. Whereas Li et al. [47] and Ylilauri et al. [49] used clinically meaningful cognitive endpoints and more extensive covariate adjustment, including adjusting for dietary intake of energy and food groups, which Huang et al. [42] did not adjust for but which may influence cognitive function [52]. Reverse causation may also explain this finding, as dietary changes in individuals experiencing early cognitive symptoms could influence intake reporting. Previous literature has identified this bias in dietary studies of older adults [[53], [54], [55]].

Findings for verbal memory and learning were similarly mixed. Most studies reported null associations, but two cross-sectional analyses [46,48] found that middle aged to older adults (aged over 50 years) with moderate or higher egg intake had slower rates of memory decline or higher scores on memory tasks. These findings align with mechanistic hypotheses and some supplementation trials indicating that choline supports hippocampal-dependent memory processes through its role in acetylcholine synthesis [17,56], and that phospholipids can modulate neurotransmitter receptors [24]. Furthermore, phospholipid-enriched diets may also improve memory, by contributing to acetylcholine production through the metabolism of phosphatidylcholine, and increasing uptake of dietary choline [57]. Intervention trials have suggested memory enhancement following phospholipid supplementation, including in a 12-week treatment of 300 mg or 600 mg daily phospholipid supplements (equivalent to 1–2 eggs per day) in elderly people with memory loss at baseline [58]. Additionally, phospholipids specifically derived from egg yolks have protected against memory loss in mice [59].

Verbal fluency outcomes also showed some consistency. Ylilauri et al. [49] and Huang et al. [42] reported higher verbal fluency scores among individuals with moderate egg intake (approx. 16–32 g of eggs per day, equivalent to 0.5–1 whole egg). While both studies used different tools (Ylilauri et al. [49] used the Verbal Fluency Test, which measures both semantic and phonemic fluency, Huang et al. [42] used the MoCA, which measures only phonemic fluency), and thus are incomparable, these findings are biologically plausible. Verbal fluency involves executive processes, lexical access, and working memory - functions influenced by neurotransmitter availability and synaptic plasticity [60]. Therefore, the choline and phospholipid content of eggs may have had an impact on this parameter of cognitive function. Although evidence from egg-specific trials is sparse, similar effects have been reported in observational studies on choline intake, finding that 187–399 mg per day (approximately two eggs) was associated with better verbal fluency [20]. This study used the Animal Fluency test to measure verbal fluency in adults in the USA aged over 60 years, which is comparable to An et al. [41] who used the same tool and cohort (data collected from the 2011−2014 NHANES) to measure verbal fluency in relation to egg consumption, but did not note significant results. The average daily egg intake in the latter study [41] was 34.3 g of eggs, which is less than one whole egg. Therefore, the average egg intake of individuals in this study may not have been sufficient to observe an impact of the dietary choline on cognitive outcomes.

The majority of studies examining processing speed and executive function domains reported no significant associations, with the exception of two studies suggesting possible links [44,49]. While the Trail Making Test (used by Ylilauri et al. [49]) and Digit Span Forwards (used by An et al. [44]) have been validated in healthy populations including middle aged to older adults [[61], [62], [63]], tools used by other studies were validated in different cohorts. The Symbol Digit Modalities Test (used by An et al. [44]) is widely validated in multiple sclerosis [[64], [65], [66]], and the Digit Symbol Substitution Test (used by An et al. [41]) is validated in Alzheimer’s disease but fails to separate mild cognitive impairment from cognitive decline [67,68]. Therefore, the assessments used in these studies may not be sensitive enough to detect subtle cognitive changes in healthy populations.

Visuospatial and orientation domains on the MoCA were also inconsistently linked to egg intake, with only one study [42] showing significant differences in lower intake quartiles. These outcomes are also known to vary substantially with age, education, and baseline cognitive status, all of which were variably accounted for across studies.

The only included pre-post study [40] demonstrated that regular consumption of 2 eggs per day improved reaction time in healthy young adults. These results were extracted from a randomised controlled trial that compared the effect of DHA-fortified eggs to regular eggs. Because our synthesis focused only on within-group pre-post changes in the control group (regular eggs), this finding should be interpreted cautiously and does not represent a true between-group effect of egg intake on cognitive outcomes. The original trial [40] found that DHA-fortified eggs led to greater improvements in reaction time and increased EEG power across Delta, Theta, and Alpha frequencies, compared to regular eggs. This study provides preliminary experimental evidence that egg-based delivery of DHA may influence neural activation patterns. However, the trial’s small sample, short duration, and homogeneous population (young adult males in military training) limit its generalisability.

A comparison with broader dietary pattern literature reveals that eggs are not commonly studied in isolation. Most cognitive nutrition studies focus on whole-diet approaches such as the Mediterranean or MIND diets, which emphasise nutrient-dense plant-based foods and typically limit cholesterol intake [[8], [9], [10]]. As a result, the independent role of eggs - a food high in both beneficial nutrients (e.g., choline, DHA) and historically discouraged components (e.g., cholesterol) - remains unclear. The current review contributes to this knowledge gap by focusing on egg intake specifically and identifying domain-specific effects.

### 4.1 Limitations

Several limitations of this review must be acknowledged. First, heterogeneity in study design, cognitive assessments, and dietary intake measures limited the ability to perform a meta-analysis. Second, most studies were observational and thus prone to confounding and bias. While most cohort studies adjusted for key covariates, residual confounding remains possible. Third, dietary exposure was commonly measured by food frequency questionnaires or brief dietary screeners, which may not capture egg consumption with sufficient precision. Fourth, variation in the definition of intake categories (e.g., “high” or “moderate” intake) limited comparability. Finally, cognitive outcomes were assessed using both objective tests and self-report measures, introducing variability in sensitivity and specificity.

### 4.2 Implications for research

The mixed evidence presented in this review highlights several priorities for future research. First, more randomised controlled trials are needed to determine the causal effects of egg intake on cognitive outcomes, with sufficient sample size, duration, and diverse populations. Second, harmonised definitions of egg intake (e.g., grams/day or number of eggs/week) would improve comparability across studies. Third, future work should include validated cognitive batteries that are domain-specific and sensitive to early cognitive change. Fourth, investigations into the role of egg-derived nutrients - such as choline, DHA, and phospholipids - should be supported by biomarker data to better elucidate mechanistic pathways. Finally, exploration of the gut–brain axis as a mediator of egg effects on cognition is warranted, given emerging evidence from separate studies demonstrating gut microbiota modulation by egg-derived components [[69], [70], [71]].

## 5 Conclusions

This systematic review identified preliminary observational evidence that moderate habitual egg consumption may be associated with better cognitive performance, particularly in memory and verbal fluency domains, and reduced risk of cognitive impairment in adults without chronic disease. However, findings were inconsistent, and the overall evidence base remains limited in both quantity and quality. Further rigorous studies, especially well-powered randomised controlled trials, are required to determine whether egg consumption contributes to cognitive resilience and to clarify dose–response relationships. These efforts will help determine whether eggs can be recommended as part of evidence-based dietary strategies to support cognitive function in aging populations.

## CRediT authorship contribution statement

NS, JRB, NJK, CJT, CM: developed the protocol.

NS, JRB: eligibility screening.

NS, EC, JRB, NJK, CJT: data extraction.

NS, NJK: risk of bias assessments.

NS, JRB, NJK, CJT, CM: interpreted the data.

NS: wrote the initial manuscript.

NS, JRB, NJK, CJT, EC, CM: critically revised the manuscript.

All authors have read and approved the final manuscript.

## Consent for publication

Not applicable.

## Ethical approval

Not applicable.

## Funding

NS was supported by a PhD Scholarship partly funded by Australian Eggs Ltd (GROW005). The funder was not involved in the study design, collection, analysis, interpretation of data, the writing of this article or the decision to submit it for publication. JRB is supported by a National Health and Medical Research Council Emerging Leadership Fellowship (APP2025943).

## Declaration of Generative AI and AI-assisted technologies in the writing process

The authors declare that generative AI was not used in the planning, writing, nor editing of this manuscript. All content is the original work of the authors and no part of the work was generated or assisted by AI technologies.

## Availability of data and materials

Data used in this review is available from the corresponding authors upon reasonable request.

## Declaration of competing interest

Nil.
