# Supplementary material for: Egg intake and cognitive function in healthy adults: A systematic review of the literature
Source: J Nutr Health Aging. 2025 Oct 7;29(12):100696. doi: 10.1016/j.jnha.2025.100696 (PMC12538690; doi:10.1016/j.jnha.2025.100696)
Supplement: Supplementary file 1 [file mmc1.docx]

**Supplementary Materials**

**Supplemental Table 1. Search strategy, MEDLINE**

| **#** | **Query** | **Results from 18 Feb 2025** |
| --- | --- | --- |
| 1 | (egg* adj3 (ingest* or intake or eat* or consum*)).ti,ab,kf. | 3,038 |
| 2 | exp Cognition/ or exp Learning/ or exp Thinking/ or exp Perception/ or exp Psychophysiology/ | 1,919,068 |
| 3 | exp Executive Function/ | 21,684 |
| 4 | [inhibition.mp](http://inhibition.mp/). [mp=title, book title, abstract, original title, name of substance word, subject heading word, floating sub-heading word, keyword heading word, organism supplementary concept word, protocol supplementary concept word, rare disease supplementary concept word, unique identifier, synonyms, population supplementary concept word, anatomy supplementary concept word] | 992,912 |
| 5 | (memory or interoception or decision-making or decision making or problem-solving or problem solving or reaction-time or reaction time or processing-speed or processing-speed or risk-taking or risk taking).mp. [mp=title, book title, abstract, original title, name of substance word, subject heading word, floating sub-heading word, keyword heading word, organism supplementary concept word, protocol supplementary concept word, rare disease supplementary concept word, unique identifier, synonyms, population supplementary concept word, anatomy supplementary concept word] | 743,997 |
| 6 | 2 or 3 or 4 or 5 | 3,169,316 |
| 7 | 1 and 6 | 146 |
| 8 | limit 7 to humans | 110 |
| 9 | limit 8 to animals | 27 |
| 10 | 8 not 9 | 83 |
| 11 | limit 10 to "review articles" | 3 |
| 12 | 10 not 11 | 80 |
| 13 | limit 12 to english language | 73 |
| 14 | limit 13 to yr="2023 -Current" | 10 |

**Supplemental Table 2. Funding sources of included studies**

| **First author (year of publication)** | **Funding source** |
| --- | --- |
| An (2019) | State key program of the national natural science foundation of China |
| An (2021) | American Egg Board’s Egg Nutrition Center. |
| Bishop (2019) | American Egg Board’s Egg Nutrition Center. |
| Huang (2021) | The Ministry of Finance of China, the National Key R&D Program of China, Precision Medicine Project–Cohort Study on Nervous System Diseases (2017YFC0907700), Community-based Cohort Study on Nervous System Diseases (2017YFC0907701). |
| Igbinigie (2024) | This research received no external funding |
| Kaewsutas (2016) | Alltech Inc. for the microalgae (All-G-RichTM) and lab analysis costs of the eggs. CP research farm for supplying the layers and feed to produce the control and DHA-enriched eggs. |
| Lee (2021) | The Biopsychosocial Religion and Health Study and parent  Adventist Health Study-2 study were funded by the National  Institute on Aging (1R01AG026348) and the National Cancer  Institute (1U01CA152939), respectively. The present analysis  was funded by the American Egg Board (AEB) |
| Li (2022) | This research was supported by Zhejiang Provincial Natural Science Foundation of China (LQ19H260001), Zhejiang Provincial Public Welfare Technology Application Research Project of China (LGF21H260002), and Medical Health Science and Technology Project of Zhejiang Provincial Health Commission (2021KY619). |
| Sukik (2022) | National Institute for Nutrition and Health, China Center for Disease Control and Prevention, Carolina Population Center (P2C HD050924, T32 HD007168), the University of North Carolina at Chapel Hill,  the NIH (R01-HD30880, DK056350, R24 HD050924 and R01-  HD38700) and the NIH Fogarty International Center (D43  TW009077, D43 TW007709), China-Japan Friendship Hospital, Ministry of Health for support for CHNS 2009, Chinese National Human Genome Center at Shanghai since 2009, and Beijing Municipal Center for Disease Prevention and Control since 2011. |
| Ylilauri (2017) | The KIHD project was funded by a large number of research  grants given to JTS. JTS is the chief executive officer of MAS-Metabolic Analytical Services Oy. |
| Xu (2022) | This study was supported by the National Social Science Foundation  of China (Grant No. 18ZDA085) |
